# Female genital mutilation: a systematic review of research on its economic and social impacts across four decades

**DOI:** 10.3402/gha.v9.31489

**Published:** 2016-10-04

**Authors:** Emmanuel Kabengele Mpinga, Aurélie Macias, Jennifer Hasselgard-Rowe, Ngianga-Bakwin Kandala, Tshimungu Kandolo Félicien, Henk Verloo, Ngoyi K. Zacharie Bukonda, Philippe Chastonay

**Affiliations:** 1 Department of Community Health and Medicine, Faculty of Medicine, Institute of Global Health, University of Geneva, Geneva, Switzerland; 2 Department of Mathematics, Physics and Electrical Engineering, Faculty of Engineering and Environment, Northumbria University, Newcastle upon Tyne, UK; 3 Division of Epidemiology and Biostatistics, School of Public Health, University of the Witwatersrand, Johannesburg, South Africa; 4 Department of Epidemiology and Public Health Sciences, The Institut Supérieur des Techniques Médicales, Kinshasa, Democratic Republic of Congo; 5 University of Applied Sciences of Western Switzerland, Sion, Switzerland; 6 Department of Public Health Sciences, Wichita State University, Wichita, KS, USA; 7 Department of Medicine, University of Fribourg, Fribourg, Switzerland

**Keywords:** female genital mutilation, systematic review, Africa, medical and psychological consequences, prevalence and ethics, socioeconomic consequences

## Abstract

**Background:**

Global efforts to end female genital mutilation (FGM) have intensified in recent decades because of the rising awareness that such a practice is an act of extreme violence against women and girls. Articles on FGM have been published highlighting the combined efforts of international and non-governmental organizations, governments, as well as religious and civil society groups to end the practice. However, the consequences of this research are not well known, and it seems that the socioeconomic aspects of the practice are underreported.

**Objective:**

This review aims to characterize over a 40-year period the scientific output on the consequences of FGM in African countries, the most affected region known for the high prevalence of FGM, and review data on the socioeconomic consequences of the practice.

**Design:**

A systematic review of literature was done, looking at the following databases: PubMed, Embase, CINAHL, BDSP, Web of Science, PsycINFO, FRANCIS, Sociological Abstracts, WHOLIS, RERO, and SAPHIR. The analysis was limited to articles concerning the African continent, published in English and French, from January 1, 1972, to December 31, 2011.

**Results:**

One hundred ninety-eight articles were reviewed. More than half of the articles were published during the last decade of the study period. The majority of papers were published in biomedical journals (64.1%). Most studies looked at Africa as a region (33.3%). Nigeria was the single country most investigated (19.2%), followed by Egypt (10.6%). Most first authors were affiliated to non-African countries (60.6%): among them 21.2% were US-based, 4% were from African institutions, and 16.2% from Nigeria.

The medical and psychological consequences (51.5%) and the prevalence and ethics of the practice (34.4%) were the most frequently investigated topics. The socioeconomic consequences were addressed in a minority of the papers (14.1%): they were classified into direct economic consequences (2.5%), school attendance (1%), marriageability (2%), sexual and marital consequences (3.5%), fertility (2.5%), domestic violence (1%), and discrimination (1.5%).

**Conclusions:**

The publication of articles on the consequences of FGM is increasing, but there is little research on the socioeconomic consequences of the practice. More scientific data focusing on this dimension is necessary to strengthen prevention, advocacy, and intervention campaigns.

## Introduction

More than 200 million women and girls alive today have been victims of female genital mutilation (FGM)^1^ in Africa, the Middle East, and Asia (1). The practice is also encountered in Europe and North America mostly in immigrant communities from countries where the prevalence is high. The battle against this phenomenon has been enhanced by two complementary movements: first, the development of human, child, and women's rights (with the Convention on the Right of the Child, Convention on All Forms of Discrimination against Women, and the Declaration on the Elimination of Violence against Women) (2); and second, the growing interest in reproductive health, and maternal and neonatal mortality (3).

Over the past four decades, progress has been reported in the following areas:

From a legislative perspective, 16 of 29 African governments of states where FGM is prevalent have adopted laws against the practice in 2009 (4). At present, 18 countries have adopted a national legislation against FGM (5). Twenty-five of these 29 countries have signed and ratified the Protocol to the African Charter on Human and People's Rights on the Rights of Women in Africa that was adopted by the African Union in 2003 (6). This protocol requires States Parties to prohibit FGM through legislative measures backed by sanctions. Furthermore, 14 concerned African countries have ratified the Convention on the Elimination of all Forms of Discrimination against Women (5, 7).

At an operational level, the World Health Assembly passed a resolution (WHA61.16) in 2008 on the elimination of FGM, emphasizing the need for action in health, education, finance, justice, and women's affairs (8). In March 2009 and June 2012, the European Parliament adopted resolutions on combatting/ending FGM (9, 10). In December 2012, the 194 UN Member States approved a General Assembly resolution, ‘Intensifying Global Efforts for the Elimination of Female Genital Mutilations’, calling on all countries to enact legislation banning FGM, as well as raising awareness and allocating sufficient resources to protect and support women and girls (11).

From an organizational approach, since 2007, the United Nations Population Fund (UNFPA) and the United Nations Children's Fund (UNICEF) are part of a joint United Nations program designed to eliminate this practice within a generation (12). In 2010, the END FGM European Network (set up by 11 European non-government organizations) addressed the negative impact FGM has on the Millennium Development Goals (MDGs) (13). Among the 17 Sustainable Development Goals (SDGs), the international community is committed to achieving gender equality and empowering all women and girls by eliminating all harmful practices such as child marriage, early or forced marriage, and FGM (14). Another recent notable movement is the joint Collaboration Strategy on Elimination of Harmful Traditional Practices established in 2013 between the Inter-African Committee on Traditional Practices, the African Union Commission, UN Economic Commission for Africa, UNICEF, UNFPA, and the African Committee of Experts on the Rights and Welfare of the Child (15).

On the research and training aspects, journals, such as the *BJOG: An International Journal of Obstetrics and Gynaecology* amongst others, have substantially contributed to the publication of FGM-related articles. Many of these articles are aimed at medical professionals and provide recommendations on how to manage victims of the practice. The UN has also developed a special program of research, development, and research training in human reproduction (HRP), first established in 1972, that addresses priorities for research to improve sexual and reproductive health (16).

This rise in international concern for FGM has led to numerous publications. However, the characteristics of this scientific output in terms of volume, main authors, means of dissemination, and research themes have not been studied thoroughly. Moreover, this literature has primarily concentrated on the prevalence of FGM, the medical consequences and its management, and only very rarely on the social and economic implications of this practice. This lack of information deprives the scientific planners, political decision makers, and community leaders of significant data that would facilitate advances in FGM prevention.

Our systematic review of literature aims to analyze the characteristics of the published papers on FGM in Africa over the last 40 years and evaluate what has been investigated in terms of the socioeconomic consequences of the practice. This will enable us to define the needs for future research in this field.

## Methods

### Data

**Fig. 1:**
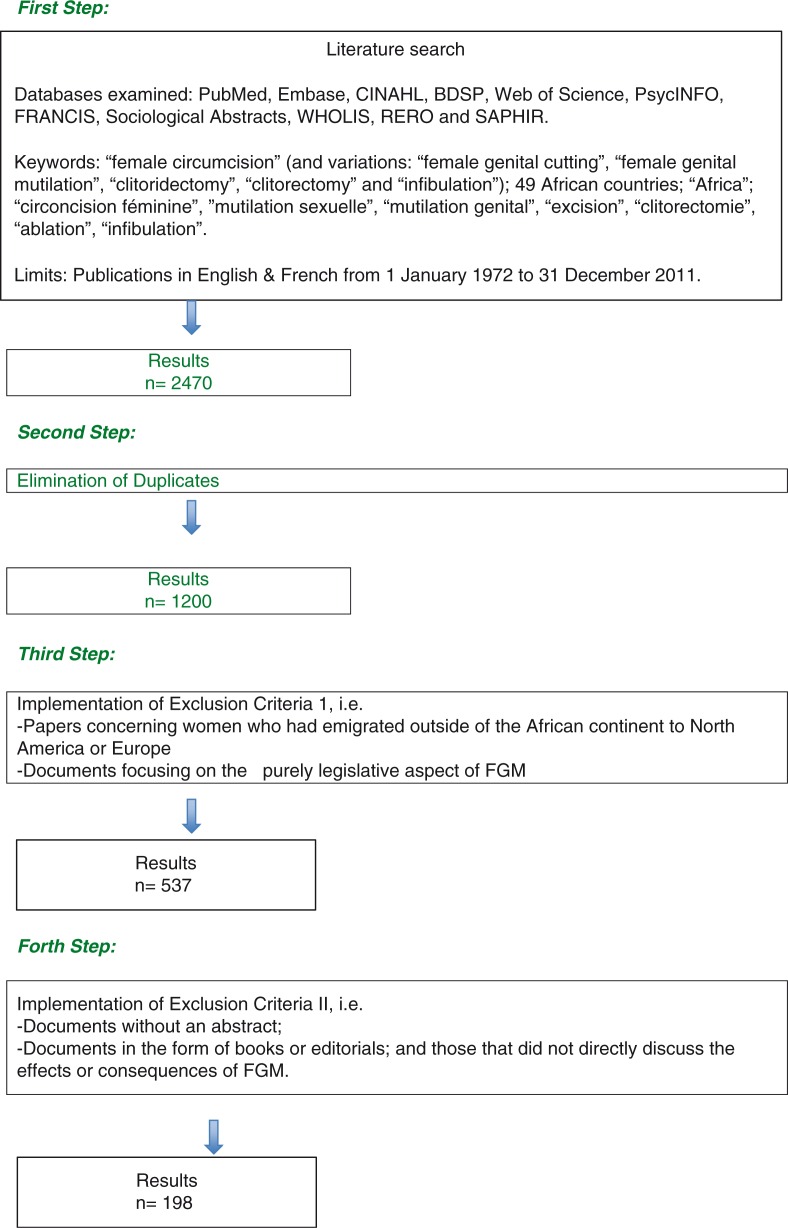
Methodology of the research strategy and data selection

This systematic review of the literature focused on the articles concerning the consequences of FGM, published during the 40-year period from January 1, 1972, to December 31, 2011. The databases used to identify these articles were PubMed, Embase, CINAHL, BDSP, Web of Science, PsycINFO, FRANCIS, Sociological Abstracts, WHOLIS, RERO, and SAPHIR (Fig. 1). This broad range of research allowed including journals covering both the biomedical and social sciences. The research was limited to articles in English and French and the geographical area studied was limited to countries of the African continent.

The keywords used for the systematic research were ‘female circumcision’ and included the variations of the same semantic field: ‘female genital cutting’, ‘female genital mutilation’, ‘clitoridectomy’, ‘clitorectomy’ and ‘infibulation’. In order to cover the entire African continent, names of 49 African countries and the word ‘Africa’ were also used as research keywords. The recognized states in Africa that were not used as keywords are South Sudan (which declared its independence in 2011); the islands of The Comoros, Mauritius, and São Tomé and Principe; the disputed territory of Western Sahara; and the self-declared independent state of Somaliland. The keywords used for the databases in French were ‘circoncision féminine’, ‘mutilation sexuelle’, ‘mutilation genital’, ‘excision’, ‘clitorectomie’, ‘ablation’, ‘infibulation’, as well as all the African countries in their French denomination. The detailed research strategy can be found in Annex 1.

### Inclusion and exclusion criteria

The initial research resulted in 2,470 references, which after the elimination of duplicates came down to 1,200. An independent manual screening by two of the authors (EKM and NBK) was then done in order to exclude all papers that concerned women who had emigrated outside of the African continent to North America or Europe and documents that concentrated only on the purely legislative aspect of the practice. Of the 537 references remaining, those without an abstract, editorials, and books, as well as those which did not directly discuss the effects or consequences of FGM were then excluded independently by EKM and NBK, i.e. 339. The final list consisted of 198 articles (Fig. 1).

### Analyzing procedure

The articles of the list were then analyzed and organized in an Excel database into different columns that included the following: year of publication, country of affiliation of the principal author (first author), type of journal, study design, study setting, main epidemiological results, main medical results, socioeconomic results, conclusion, and language. Concerning the category of type of journal, articles were classified into biomedical and care journals, epidemiology and public health journals, mixed journals (medicine plus other), journals of social sciences, and other for those that did not fit into any of the previous categories. As for the type of study design, the documents were separated between cross-sectional, cohort, case–control, qualitative studies (which included interviews), case series, social analyses (which included sociopolitical, legal, and anthropological studies), economic studies, simple reviews, systematic reviews, and others (for educational recommendations and reports of conferences). For the latter, educational recommendations corresponded to the articles aimed at nurses, midwives, and doctors, which contained recommendations on their attitudes as medical professionals.

With regard to the authorship countries, they were categorized given the institutional belonging of the main author. Often, it was the author responsible for the correspondence. As for the study setting, this refers to the geographical region and type of population the study concentrated on. In cases where the results and discussion concerned all women suffering from FGM mutilation in general but no specific country has been studied, the classification was *Africa as a region*. If the study was carried out in more than one country, it was tagged as *Multi-site in Africa*.

Finally, the articles were classified according to their main research topic. Special attention was given to articles focusing on the socioeconomic consequences of this practice, both directly and indirectly, and what their results were. These were separated depending on how they approached the socioeconomic question: directly quantified economic costs, school attendance, sexual and marital consequences, fertility, domestic violence, discrimination, and marriageability.

The research strategy and data selection process are presented in Fig. 1.

## Results

The results, summarized in Tables (1–6), are based on 198 articles concerning the consequences of FGM over a 40-year period (1972–2011); 90.4% of the articles are in English and 9.6% in French. More than half of the articles were published over the last decade of study, i.e. 52.8% (*n*=104) between 2002 and 2011. Between 1972 and 1981, the published articles represent 6.6%; between 1982 and 1991, 10.7%; and between 1992 and 2001, 29.9% (Table 1).

**Table 1:** Years of publication

Year	Number of publications	%
2002–2011	104	52.5
1992–2001	59	29.8
1982–1991	21	10.6
1972–1981	14	7.1
Total	198	100

In Table 2, the articles are listed according to their study design. Cross-sectional studies represent the most frequent study design (with 32.3%, *n*=64). The second most commonly used study design is social analysis, representing 20.2% of the studies. This category includes the sociopolitical, legal, and anthropological studies. The design that follows in terms of quantity is the simple review (i.e. non-systematic nor exhaustive review) making up 14.1%. Cohort studies represent 8.1%, case series 7.1%, and case–control 2% of the articles. Only 1.5% (three articles) were purely economic studies. Systematic reviews were the least utilized design representing 1% (*n*=2). The category ‘other’ was created for the educational recommendations and reports of conferences, which together make up 8.6% of the total studies analyzed. Overall, epidemiological studies, which include cross-sectional, cohort, and case–control studies combined, make up 42.4% of the examined articles.

**Table 2:** Study designs

Designs	*n*	%
Cross-sectional	64	32.3
Cohort	16	8.1
Case-control	4	2
Qualitative studies	10	5.1
Case series	14	7.1
Social analyses	40	20.2
Economic studies	3	1.5
Simple reviews	28	14.1
Systematic reviews	2	1
Other (educational recommendations, reports of conferences)	17	8.6
Total	198	100

In Table 3, the categories of journals where the reviewed articles on FGM were published are listed. The majority of articles published were biomedical and care journals (64.6%), which include biomedicine journals (59.6%) and nursing journals (4.5%); 15.7% of the articles reviewed were published in epidemiology and public health journals; 11.6% in mixed journals comprising journals of medicine and social sciences, of health and human rights, and of medicine and law; 7.6% in social sciences journals; finally, 1% of papers were published in the category ‘others’ (e.g. an engineering journal, an environmental journal).

**Table 3:** Categories of journals

Journals	*n*	%
1. Biomedical and care journals	127	64.1
Biomedicine	118	59.6
Nursing	9	4.5
2. Epidemiology and public health	31	15.7
3. Mixed journals	23	11.6
Medicine and social sciences	18	9.1
Health and human rights	4	2
Medicine and law	1	0.5
4. Journals of social sciences	15	7.6
Mixed social sciences	9	4.5
Human rights	4	2
Economics	1	0.5
Ethics	1	0.5
5. Other	2	1
Engineering	1	0.5
Environment/sustainability	1	0.5
Total	198	100

Table 4 shows the classification of papers depending on the study settings. Most of the articles discussed the situation of women in Africa as a region (33.3%), without concentrating on a specific study area and 8.1% of the articles concerned multiple sites in Africa. Apart from this, the country in which the largest number of studies took place was Nigeria with 19.2%, followed by Egypt (10.0%), Sudan (5.6%), and Somalia (4.1%); Burkina Faso, Ethiopia, and Kenya with 2.5% each; Tanzania, Eritrea, and Ghana with 1.5% each; Chad and Gambia with 1% each; and Cameroon, Guinea, Ivory Coast, Mali, Niger, and Senegal with only one article each (0.5%).

**Table 4:** Study settings

Study settings	*n*	%
Africa as a region	66	33.3
Nigeria	38	19.2
Egypt	21	10.6
Multi-site in Africa	16	8.1
Sudan	11	5.6
Somalia	8	4.1
Burkina Faso	5	2.5
Ethiopia	5	2.5
Kenya	5	2.5
Tanzania	3	1.5
Eritrea	3	1.5
Ghana	3	1.5
Chad	2	1
Gambia	2	1
Others^a^	10	5.1
Total	198	100

a Others: Cameroon, Guinea, Ivory Coast, Mali, Niger, Senegal.

Regarding the main author's country of affiliation defined according to the location of the institution hosting the main author, authors from the United States published the most papers (21.2%). The second biggest contributor was Nigeria with 16.2%. This was followed by the UK (9.1%), Egypt (7.6%), international organizations (6.6%), France (4.1%), and Sweden (3.5%). Ethiopia, Kenya, Saudi Arabia, and Sudan all contributed equally with 2%, and Switzerland with 1.5%. Finally, there are 23 other countries that are responsible for 2 or less articles each and together they make up to 18.2% of the total publications. These countries are listed in Table 5. All in all, the authors affiliated with African countries made up 39.4%, compared to 60.6% affiliated with non-African countries.

**Table 5:** Country affiliation of main author

Authorship countries	*n*	%
USA	42	21.2
Nigeria	32	16.2
UK	18	9.1
Egypt	15	7.6
International organizations	13	6.6
France	8	4.1
Sweden	7	3.5
Ethiopia	4	2
Kenya	4	2
Saudi Arabia	4	2
Sudan	4	2
Switzerland	3	1.5
Others^a^	36	18.2
N/A	8	4
Total	198	100

a Others: Australia, Belgium, Burkina Faso, Cameroon, Canada, Cuba, Denmark, Gambia, Germany, Ghana, Israel, Italy, Kuwait, Mali, Mexico, the Netherlands, Norway, Oman, Poland, Senegal, South Africa, Spain, Pakistan.

Concerning the research issues, 51% of the articles explored the extensive list of short- and long-term medical and psychological consequences on women, as their main research topic (Table 6). Thirty-four percent of the articles focused primarily on the prevalence of FGM, and some on the ethical aspects related to the practice. Only 14% of the published articles focused on the socioeconomic consequences of FGM. As seen in Tables 6 and 2.5% quantify the economic consequences; 1% mention the effect on school attendance and productivity; 3.5% focus on the sexual consequences of FGM by studying the sexual dysfunction and marital problems deriving from it; 2.5% explore the question of the impact on fertility; and 1% describes the association of the victims of the practice with domestic violence. With regard to social status, 1.5% developed the topic of discrimination of uncircumcised women, and 2% studied the difficulty of marriageability and the bridal market. All the 28 studies (17–44) are summarized in Table 7.

**Table 6:** Research themes

Research themes	*n*	%
1. Medical and psychological consequences	102	51.5
2. Prevalence and ethics	68	34.4
3. Socio-economic consequences	28	14.1
Direct economic consequences	5	2.5
School attendance	2	1
Sexual and marital consequences	7	3.5
Fertility	5	2.5
Domestic violence	2	1
Discrimination	3	1.5
Marriageability	4	2
Total	198	100

**Table 7:** Socio-economic findings related to FGM

Direct quantified economic consequences	1. Victims of type 3 FGM have a shortened life expectancy. Annual costs of FGM-related obstetric complications in six African countries represent $3.7 million or 0.1-1% of government spending on health for women aged 15–45 years. Global loss of life years of FGM is estimated to up of 2.8 million life years (17).
	2. In a Nigerian University Hospital study the duration of necessary clinical follow-up due to medical complications was 13 months (18).
	3. At a Nigerian Medical Center the average management cost of medical complications of FGM per victim was $120 (19).
	4. At a Maternity Hospital in Somalia the mean number of hospitalization days per victim due to FGM complications was 16.5 days (20).
	5. Obstetric and gynecological operations to treat direct complications of female circumcision in a Sudanese Teaching Hospital represented 7% of the total number of operations (21).
School attendance	1. Immediate marriage with no return to school after the FGM procedure has been reported from Kenya (22).
	2. Female circumcision contributes to high school dropout (23).
Sexual and marital consequences	1. In Guinea FGM did not affect the likelihood of premarital sex nor marriage (24).
	2. In Egypt marital/sexual problems (dyspareunia, loss of libido, failure of orgasm and husband's dissatisfaction) was higher among circumcised women (25).
	3. In Egypt men perceived FGM as possibly having negative effects on women's sexual response (26).
	4. In a study implemented in 5 medical centers in Egypt 68.9% of circumcised women reported having sexual problems, 31.5% suffered from dyspareunia, 49.6% had decreased sexual desire, 36% had difficulties with arousal and 16.9% had anorgasmia (27).
	5. At 3 Nigerian hospitals, FGM was shown as not attenuating the sexual arousal of women (28).
	6. In a Nigerian study being circumcised did not lead to early sexual experiences (29).
	7. In Egypt a study among 250 circumcised women, the women reported: vaginal dryness during intercourse(48.5%), lack of sexual desire (45%), less frequency of sexual desire per week (28%), less initiative during sex (11%), less pleasure from sex (49%), less orgasms (39%), less frequency of orgasm (25%), difficulty reaching orgasm (60.5%) (30).
Fertility	1. FGM is associated with and may contribute to increase the number and ratio of births of male boys (Odds Ratio = 1.019; 95% C.I.=1.007, 1.032) as shown in a study on 413,384 births from 22 African countries (31).
	2. Infertility rate in infibulated women can be as high as 30% (32).
	3. In Egypt FGM type III has been associated with infertility in representative samples of women (33).
	4. In Sudan circumcision did not lead to impaired fertility in married women, except for higher prevalence of primary infertility among those who had undergone Pharaonic (Type 3) or intermediate (Type 2) circumcision (34).
	5. Female circumcision was not associated with increased infertility nor with reduced fertility in studies from the Central African Republic, the Ivory Coast and Tanzania (35).
Domestic violence	1. In a study from Egypt holding positive beliefs of FGM practice was associated with maternal physical violence (69.8% had hit their children during the year prior to the survey) (36).
	2. In a study from Egypt circumcised women were 7.5 times more likely to accept that husbands have the right to beat their wives (37).
Discrimination	1. The major deterrent to marriage between men from circumcising families and uncircumcised women is the hostility and discrimination an uncircumcised woman faces among circumcised women (38).
	2. In a study outpatients of a Nigerian hospital, stigmatizing attitudes toward the uncircumcised women were reported: 74% said they are promiscuous, 49% said they are shameful, 14% cursed/outcast, 66% would not recommend them for marriage (39).
	3. In Nigeria uncircumcised women were less eligible as wives and they were ostracized by women themselves (40).
Marriageability	1. From the Southern African Development Community region it is reported that women who have not undergone the practice may find it difficult to get husbands (41).
	2. The expectation that FGM leads to better marital outcomes is enough to perpetuate the practice as a social norm, even though the value of it within the marriage might be very low (42).
	3. Informants from Egypt characterized FGM as a prerequisite for marriage, enabling girls to acquire a social identity, economic security, and some measure of familial authority in a patrilineal society. A bride who proves her virginity receives material benefits, social approval and preserves the honor of her family (43).
	4. Only for a gabar gudban (closed woman) as opposed to the term buriya gab (woman with clitoris) does the father receive a good bride price (yarad), which contributes to the economic prosperity of the village (44).

## Discussion

Over the years, a clear trend indicating an increase in the number of published articles concerning the consequences of FGM can be seen. This does not come as a surprise because of the increase in global attention this issue has received over the last decade with the creation of, for example, the International Day of Zero Tolerance to FGM in 2003 by the UN (45), as well as many other movements discussed in the *Introduction* section of this paper.

Most studies were epidemiological studies published in biomedical and care journals. Only about a sixth of the articles focused on the socioeconomic consequences of the practice, hence the small number of publications in Journals of Social Sciences, which are more likely to consider socioeconomic context rather than consequences of FGM. Yet, even epidemiological studies might be difficult to correctly interpret, because most are based on the perception of victims which might be heavily influenced by the cultural environment. Furthermore, data reporting prevalence levels might also suffer from inaccuracies because of the difficulties of collecting data in field studies. It should also be mentioned that quantitative research methods are not best suited for exploring such a sensitive issue like FGM. One should keep in mind in this regard the value of qualitative research approaches which allow more precisely exploration of the cultural, social, and psychological aspects of FGM.

As discussed in the Methods section, one of the inclusion criteria was studies on populations living in Africa. Thus, all articles concerning the African immigrants in the USA and Europe were eliminated. Nevertheless, non-African researchers/countries have authored the majority of the papers. This phenomenon has already been studied by Tijssen in a paper where he shows that Africa's contribution to global knowledge production has declined. This can partly be explained through limited access of African researchers to modern information and communication technology facilities and to a lack of research funds (46). Furthermore, the immigration of people from the African continent to Europe and North America has pushed European and North American countries to fund research on FGM. According to a nationwide prevalence study released in February 2015 by the US Population Reference Bureau up to 507,000 women and girls living in the US are at risk of or have undergone FGM (47), twice as much as the estimates of 2000 (228,000) (48). Western countries had to adapt their legislative framework, because of locally performed FGM: indeed, there have been convictions for it in France and Switzerland (49).

Only one study (17) was found to address the economic costs as its main research question. This study describes the obstetric costs of FGM in terms of costs to the medical system in parity dollars, percentage of government expenditure, and loss of life-years for the affected women (15). Furthermore, there are five studies that investigate the economic burden of FGM on the health system, even if it is not their main research question. The economic burden is measured by referring to the extended follow-up periods (18), the average cost of management of FGM-related complications in a particular hospital (19), the number of hospital days of patients (20), and the percentage on the hospital's work dedicated to the consequences of FGM (21).

In the description of school attendance, no real quantification is given in terms of school days missed (22). Considering sexual and marital consequences of FGM, two studies report no effect of FGM on the earliness of sexual relations (24, 29). Five studies describe the negative impact of FGM on sexual life after marriage (25–27, 30). Regarding fertility most studies report a link between FGM and infertility (32–34), but an absence of any association has also been reported (35). Concerning domestic violence, discrimination, and marriageability, the results from the different studies are concordant, but they lack substantial quantitative data to support the extent of their claims.

As a matter of fact, none of these studies on the socioeconomic consequences of FGM are large-scale surveys, which somewhat might lessen the weight of the reported data. But, as mentioned above, valuable relevant information might only be apprehended through qualitative research methods.

The economic burden resulting from the management and treatment of the medical complications related to FGM have been reported in several studies of our systematic review, also mentioning that it might contribute to the underdevelopment of a country. However, these studies focus more on the medical aspects of the FGM-related consequences, not quantifying its economic effects nor exploring its social consequences. Some of these studies also mention the need to train health professionals in the field of FGM. Magoha (50) insists that physicians require specific training, be it general practitioners, pediatricians, proctologists, obstetricians, or plastic surgeons. Furthermore, psychiatrists, psychologists, and social workers need also to be trained in order to include all aspects of the management of FGM. Managing medical complications and training professionals clearly has a price, but this issue is only briefly mentioned in the reviewed articles.

Considering the limits of our review, let us point out that 339 documents identified through the literature search with keywords have not been included: these papers were either editorials, books, comments or articles without an abstract or paper that did not directly discuss the effects or consequences of FGM. Thus we cannot guarantee that valuable information has not been lost in the process. Furthermore, we limited our review to the FGM issue in Africa, although FGM practices are also present in other countries, notably in Asia and the Middle East. In Yemen for instance, the prevalence of FGM in girls and women aged 15–49 is 23% (51).

Our review was limited to English and French articles, *de facto* excluding publications in regional languages. However considering that French and English are official languages in the vast majority of the countries of the African continent, the lost information might be marginal. Our review also underestimates the contribution of books to the topic, as we excluded book reviews and chapters of books. Finally, due to the established 40-year study period of the review, it cannot be stated whether publications after 2011 demonstrate an increase of interest in the socioeconomic aspects of this field or not. A further limitation is the exclusion of surveyed articles from the recent period after 2011. Finally we had no access to doctoral theses, nor to articles published in local scientific journals not listed on electronic data bases accessible via Internet.

## Research perspectives and recommendations

Despite certain limitations, our review may contribute by calling attention to the lack of data in this field, and suggest relevant research approaches, such as household surveys.

This study suggests that:

- There is a need to extend the research to countries where the prevalence of FGM is highest, and thus the consequences greater. A majority of country studies reviewed focused on Nigeria, where there is a 25% prevalence of FGM (52), but we found no study on Djibouti or Sierra Leone where the estimated prevalence of FGM is as high as 90% (52).
- Medical acute and long term complications are well-studied and frequent, yet their effect on life expectancy is not.
- There is little information on costs of primary health care providers consulted before the patients get referred to secondary or tertiary health centers.
- There is insufficient data on the repercussions on school attendance. No quantitative data was found educational level attended by cut versus non-cut girls.
- There is a lack of data on the possible impact of FGM on employment. We found no investigation on the income of circumcised women versus the one of uncircumcised women, which would help draw conclusions on productivity loss.
- There is a lack of research on the effect of FGM on families as a whole. All the studies acknowledge consequences on the direct victims of FGM. Some report consequences on the partners, but none in our review has investigated the possible effects of FGM on the mothers, the children or the family structure.

## Conclusion

FGM is human rights violation that affects health. Therefore, it may seem more natural to investigate its medical complications and to initiate an ethical debate on FGM rather than to explore its economic dimensions. Through our literature review, the lack of information on the socioeconomic consequences of FGM becomes obvious. The number of studies published over a 40 years period concerning the prevalence of FGM and its medical complications is quite impressive: the evidence is conclusive and unarguable.

Although some governments have initiated prevention programs and the civil society in many countries have legislated against FGM, its prevalence remains high in many African countries (53). Hence, the need to shift some of the research on the weight this practice has in a socioeconomic perspective, all the more because societies where FGM is prevalent continue to perceive the practice as economically advantageous by believing it increases marriageability (42).

But, with additional sound scientific data, for example, good intervention studies using appropriate methodologies, the effects of FGM on society and the economy could be even better assessed and could further support the fight against FGM. Yet not only more appropriate research could contribute to better prevent FGM and support more efficiently victims of FGM: training health worker and raising the awareness of community leaders and authorities are crucial in this context.

Surely a multi-sectorial strategy in the fight of FGM is needed, including good quality research, prevention, best intervention practices, and strong advocacy.

## Footnotes

1 The World Health Organization classifies FGM into 4 major types: Type 1: Often referred to as clitoridectomy, this is the partial or total removal of the clitoris (a small, sensitive, and erectile part of the female genitals), and in very rare cases, only the prepuce (the fold of skin surrounding the clitoris). Type 2: Often referred to as excision, this is the partial or total removal of the clitoris and the labia minora (the inner folds of the vulva), with or without excision of the labia majora (the outer folds of skin of the vulva). Type 3: Often referred to as infibulation, this is the narrowing of the vaginal opening through the creation of a covering seal. The seal is formed by cutting and repositioning the labia minora, or labia majora, sometimes through stitching, with or without removal of the clitoris (clitoridectomy). Type 4: This includes all other harmful procedures to the female genitalia for non-medical purposes, e.g. pricking, piercing, incising, scraping, and cauterizing the genital area. Deinfibulation refers to the practice of cutting open the sealed vaginal opening in a woman who has been infibulated, which is often necessary for improving health and well-being as well as to allow intercourse or to facilitate childbirth.

## Paper context

Global efforts to end female genital mutilation (FGM) have intensified in recent decades and attempts are on for a better understanding of this phenomenon and strategic approaches for its prevention and eradication. Articles on FGM have been published highlighting the combined efforts of international and non-governmental organizations, governments, as well as religious and civil society groups to end the practice. However, the consequences of this scientific production are not well known, and it seems that the socioeconomic aspects of the practice are underreported. Our systematic review of literature aims to analyze the characteristics of the published papers on FGM in Africa over the last 40 years and evaluate what has been investigated in terms of the socioeconomic consequences of the practice.

## Appendix

**Annex 1:** Details of search strategy (in French and English)

Database	Search equation
1. PubMed	(“Circumcision, Female”[Mesh] OR “Female Circumcisions”[tiab] OR “Female Circumcision”[tiab] OR “Infibulation”[tiab] OR “Infibulations”[tiab] OR “Clitoridectomy”[tiab] OR “Clitoridectomies”[tiab] OR “Clitorectomy”[tiab] OR “Clitorectomies”[tiab] OR “Female Genital Cutting”[tiab] OR “Female Genital Mutilation”[tiab] OR “Female Genital Mutilations”[tiab]) AND (“Africa”[Mesh] OR "Africa*"[tiab] OR “Algeria”[tiab] OR “Egypt”[tiab] OR “Libya”[tiab] OR “Morocco”[tiab] OR “Tunisia”[tiab] OR “Cameroon”[tiab] OR “Central African Republic”[tiab] OR “Chad”[tiab] OR “Congo”[tiab] OR “Democratic Republic of the Congo”[tiab] OR “Equatorial Guinea”[tiab] OR “Gabon”[tiab] OR “Burundi”[tiab] OR “Djibouti”[tiab] OR “Eritrea”[tiab] OR “Ethiopia”[tiab] OR “Kenya”[tiab] OR “Rwanda”[tiab] OR “Somalia”[tiab] OR “Sudan”[tiab] OR “Tanzania”[tiab] OR “Uganda”[tiab] OR “Angola”[tiab] OR “Botswana”[tiab] OR “Lesotho”[tiab] OR “Malawi”[tiab] OR “Mozambique”[tiab] OR “Namibia”[tiab] OR “South Africa”[tiab] OR “Swaziland”[tiab] OR “Zambia”[tiab] OR “Zimbabwe”[tiab] OR “Benin”[tiab] OR “Burkina Faso”[tiab] OR “Cape Verde”[tiab] OR “Cote d'Ivoire”[tiab] OR “Gambia”[tiab] OR “Ghana”[tiab] OR “Guinea”[tiab] OR “Guinea-Bissau”[tiab] OR “Liberia”[tiab] OR “Mali”[tiab] OR “Mauritania”[tiab] OR “Niger”[tiab] OR “Nigeria”[tiab] OR “Senegal”[tiab] OR “Sierra Leone”[tiab] OR “Togo”[tiab])
2. Embase	(‘female circumcision’/exp OR "Female Circumcisions":ti:ab OR "Female Circumcision":ti:ab OR "Infibulation":ti:ab OR "Infibulations":ti:ab OR "Clitoridectomy":ti:ab OR "Clitoridectomies":ti:ab OR “Clitorectomy”:ti:ab OR “Clitorectomies”:ti:ab OR “Female Genital Cutting”:ti:ab OR “Female Genital Mutilation”:ti:ab OR “Female Genital Mutilations”:ti:ab) AND (“Africa”/exp OR Africa*:ti:ab OR “Algeria”:ti:ab OR “Egypt”:ti:ab OR “Libya”:ti:ab OR “Morocco”:ti:ab OR “Tunisia”:ti:ab OR “Cameroon”:ti:ab OR “Central African Republic”:ti:ab OR “Chad”:ti:ab OR “Congo”:ti:ab OR “Democratic Republic of the Congo”:ti:ab OR “Equatorial Guinea”:ti:ab OR “Gabon”:ti:ab OR “Burundi”:ti:ab OR “Djibouti”:ti:ab OR “Eritrea”:ti:ab OR “Ethiopia”:ti:ab OR “Kenya”:ti:ab OR “Rwanda”:ti:ab OR “Somalia”:ti:ab OR “Sudan”:ti:ab OR “Tanzania”:ti:ab OR “Uganda”:ti:ab OR “Angola”:ti:ab OR “Botswana”:ti:ab OR “Lesotho”:ti:ab OR “Malawi”:ti:ab OR “Mozambique”:ti:ab OR “Namibia”:ti:ab OR “South Africa”:ti:ab OR “Swaziland”:ti:ab OR “Zambia”:ti:ab OR “Zimbabwe”:ti:ab OR “Benin”:ti:ab OR “Burkina Faso”:ti:ab OR “Cape Verde”:ti:ab OR “Cote d Ivoire”:ti:ab OR “Gambia”:ti:ab OR “Ghana”:ti:ab OR “Guinea”:ti:ab OR “Guinea-Bissau”:ti:ab OR “Liberia”:ti:ab OR “Mali”:ti:ab OR “Mauritania”:ti:ab OR “Niger”:ti:ab OR “Nigeria”:ti:ab OR “Senegal”:ti:ab OR “Sierra Leone”:ti:ab OR “Togo”:ti:ab)
3. CINAHL	((MH “Circumcision, Female”) OR TI (“Female Circumcisions” OR “Female Circumcision” OR “Infibulation” OR “Infibulations” OR “Clitoridectomy” OR “Clitoridectomies” OR “Clitorectomy” OR “Clitorectomies” OR “Female Genital Cutting” OR “Female Genital Mutilation” OR “Female Genital Mutilations”) OR AB (“Female Circumcisions” OR “Female Circumcision” OR “Infibulation” OR “Infibulations” OR “Clitoridectomy” OR “Clitoridectomies” OR “Clitorectomy” OR “Clitorectomies” OR “Female Genital Cutting” OR “Female Genital Mutilation” OR “Female Genital Mutilations”)) AND (MH "Africa+") OR TI ( Africa* OR Algeria OR Egypt OR Libya OR Morocco OR Tunisia OR Cameroon OR “Central African Republic” OR Chad OR Congo OR “Democratic Republic of the Congo” OR “Equatorial Guinea” OR Gabon OR Burundi OR Djibouti OR Eritrea OR Ethiopia OR Kenya OR Rwanda OR Somalia OR Sudan OR Tanzania OR Uganda OR Angola OR Botswana OR Lesotho OR Malawi OR Mozambique OR Namibia OR “South Africa” OR Swaziland OR Zambia OR Zimbabwe OR Benin OR “Burkina Faso” OR “Cape Verde” OR “Cote d'Ivoire” OR Gambia OR Ghana OR Guinea OR “Guinea-Bissau” OR Liberia OR Mali OR Mauritania OR Niger OR Nigeria OR Senegal OR “Sierra Leone” OR Togo) OR AB ( Africa* Algeria OR Egypt OR Libya OR Morocco OR Tunisia OR Cameroon OR “Central African Republic” OR Chad OR Congo OR “Democratic Republic of the Congo” OR “Equatorial Guinea” OR Gabon OR Burundi OR Djibouti OR Eritrea OR Ethiopia OR Kenya OR Rwanda OR Somalia OR Sudan OR Tanzania OR Uganda OR Angola OR Botswana OR Lesotho OR Malawi OR Mozambique OR Namibia OR “South Africa” OR Swaziland OR Zambia OR Zimbabwe OR Benin OR “Burkina Faso” OR “Cape Verde” OR “Cote d'Ivoire” OR Gambia OR Ghana OR Guinea OR “Guinea-Bissau” OR Liberia OR Mali OR Mauritania OR Niger OR Nigeria OR Senegal OR “Sierra Leone” OR Togo)
4. BDSP	(“Mutilation sexuelle” OU Excision OU Infibulation OU “Ablation clitoris” OU “Circoncision féminine” OU Clitorectomie OU Excision OU “Mutilation génitale féminine” OU MGF) ET (Afrique OU africain OU africaine OU africains OU africaines OU Angola OU Bénin OU Botswana OU Burkina Faso OU Burundi OU Cameroun OU Canaries OU Cap Vert OU Centrafrique OU Comores OU Congo OU “Côte d'Ivoire” OU Djibouti OU Egypte OU Erythrée OU Ethiopie OU Gabon OU Gambie OU Ghana OU Guinée OU Ile Maurice OU Kenya OU Lesotho OU Libéria OU Madagascar OU Maghreb OU Malawi OU Mali OU Maurice OU Mozambique OU Namibie OU Niger OU Nigeria OU “Océan Indien” OU Ouganda OU Rwanda OU “Sahara espagnol” OU Sénégal OU Seychelles OU Sierra Leone OU Somalie OU Soudan OU Swaziland OU Tanzanie OU Tchad OU Togo OU Zaïre OU Zambie OU Zimbabwe)
5. Web of Science, PsycINFO, FRANCIS, Sociological abstracts	(“Female Circumcisions” OR “Female Circumcision” OR “Infibulation” OR “Infibulations” OR “Clitoridectomy” OR “Clitoridectomies” OR “Clitorectomy” OR “Clitorectomies” OR “Female Genital Cutting” OR “Female Genital Mutilation” OR “Female Genital Mutilations”) AND (Africa* OR Algeria OR Egypt OR Libya OR Morocco OR Tunisia OR Cameroon OR “Central African Republic” OR Chad OR Congo OR “Democratic Republic of the Congo” OR “Equatorial Guinea” OR Gabon OR Burundi OR Djibouti OR Eritrea OR Ethiopia OR Kenya OR Rwanda OR Somalia OR Sudan OR Tanzania OR Uganda OR Angola OR Botswana OR Lesotho OR Malawi OR Mozambique OR Namibia OR “South Africa” OR Swaziland OR Zambia OR Zimbabwe OR Benin OR “Burkina Faso” OR “Cape Verde” OR “Cote d'Ivoire” OR Gambia OR Ghana OR Guinea OR “Guinea-Bissau” OR Liberia OR Mali OR Mauritania OR Niger OR Nigeria OR Senegal OR “Sierra Leone” OR Togo)
6. WHOLIS, RERO, SAPHIR	Circumcision, female, Africa, African, Mutilation sexuelle, Afrique

[MeSH]=MeSH Term [tiab]=Search in title (ti) and abstract (abstract) /exp = Emtree term :ti:ab=Search in title (ti) and abstract (abstract) MH=Subject heading TI, AB=Search in title, abstract
